# Discrepancy between self-reported intoxication and psychomotor vigilance task performance after alcohol intake: associations with BrAC, alcohol-metabolizing genotypes, and drinking tendency

**DOI:** 10.1186/s40101-026-00434-w

**Published:** 2026-06-10

**Authors:** Midori Motoi, Nakyeong Shin, Momo Hama, Yukitaro Yasuda, Takayuki Nishimura, Eigo Nishimura, Hideo Toyoshima, Yuiko Araki, Takafumi Katsumura, Yali Xia, Tomoaki Ohashi, Nariaki Kuriyama, Yuna Miyauchi, Yuki Motomura, Shigekazu Higuchi

**Affiliations:** 1 Department of Human Life Design and Science, Faculty of Design, Kyushu University, 4-9-1 Shiobaru, Minami-Ku, Fukuoka, 815-8540 Japan; 2 Graduate School of Integrated Frontier Sciences, Kyushu University, 4-9-1 Shiobaru, Minami-Ku, Fukuoka, 815-8540 Japan; 3 Graduate School of Design, Kyushu University, 4-9-1 Shiobaru, Minami-Ku, Fukuoka, 815-8540 Japan; 4 School of Nursing, Faculty of Nursing, Reiwa Health Sciences University, 2-1-12, Wajirogaoka, Higashi-Ku, Fukuoka, 811-0213 Japan; 5 Fukuoka Urasoe Clinic, BCC Building 9F, 2-12-19 Ropponmatsu, Chuo-Ku, Fukuoka, 810-0044 Japan; 6 Honda R&D Co., Ltd., 1-4-1, Chuo, Wako-Shi, Saitama, 351-0193 Japan; 7 Honda Research Institute Japan Co., Ltd., 8-1 Honcho, Wako-Shi, Saitama, 351-0188 Japan

**Keywords:** Breath alcohol concentration, Subjective intoxication, *ALDH2*, AUDIT-C, PVT, Structural equation modeling

## Abstract

**Introduction:**

The degrees of subjective intoxication (SI) and vigilance impairment following alcohol consumption vary among individuals. This variability may reflect both metabolic enzyme genotypes and drinking behavior. The purpose of this study was to examine the integrated associations among alcohol-metabolizing enzyme genotypes, drinking tendency, and acute alcohol-induced changes in breath alcohol concentration (BrAC), SI, and vigilance performance.

**Methods:**

Alcohol (0.5 g/kg body weight) was orally administered to 93 healthy adults. BrAC, SI, and the Psychomotor Vigilance Task (PVT) were measured at multiple time points between 0 and 100 min. Repeated-measures ANOVA was performed with time as the within-subject factor and *alcohol dehydrogenase 1B* (*ADH1B*) and *aldehyde dehydrogenase 2* (*ALDH2*) genotypes as between-subject factors. Additionally, using structural equation modeling (SEM), we examined how BrAC, SI, and PVT lapse rates were jointly related to genotypes and the Alcohol Use Disorders Identification Test–Consumption (AUDIT-C) score.

**Results:**

For BrAC and SI, a significant *ALDH2* × time interaction was observed. ALDH2*1/*1 participants had lower BrAC than ALDH2*1/*2 participants. Regarding PVT performance, the main effect of time was observed, but no significant main effects or interactions involving genotypes were found. Additionally, results from SEM showed that, in the 40-min model, BrAC positively predicted SI and lapse rate, and AUDIT-C negatively predicted SI but not lapse rate. *ALDH2* genotype positively predicted AUDIT-C and negatively predicted BrAC; *ALDH2* genotype also showed a significant direct negative path to SI but not on lapse rate. In the 100-min model, BrAC positively predicted SI but not lapse rate, and AUDIT-C negatively predicted SI but not lapse rate.

**Conclusion:**

*ALDH2* genotype contributed to variability in BrAC and SI but not PVT performance. In the 40-min SEM model, higher AUDIT-C predicted lower SI despite no association with BrAC or PVT lapse rate. This pattern suggests a discrepancy between self-reported intoxication and PVT performance. These findings may inform risk communication and intervention strategies to reduce alcohol-related accidents and improve health education.

**Trial registration:**

UMIN Clinical Trials Registry (UMIN-CTR), UMIN000055964, registered on 2024–10-28.

**Supplementary Information:**

The online version contains supplementary material available at 10.1186/s40101-026-00434-w.

## Introduction

Alcohol consumption poses significant public health challenges owing to its association with traffic accidents, violence, acute health damage, and chronic health consequences resulting from long-term alcohol use [1]. Acute alcohol intake rapidly impairs the psychological and behavioral functions essential for safe decision-making, including alertness, attention, and inhibitory control [2, 3]. These acute effects are well documented, and their magnitude varies markedly across individuals, even after the same alcohol dose. Such variability likely reflects influences operating at multiple levels, including biological factors (e.g., genetic background and sex) and behavioral factors. It remains unclear how these factors jointly shape subjective intoxication (SI) and vigilance impairment during acute alcohol exposure. This gap limits understanding of the mechanisms underlying risky drinking behaviors and alcohol-related harm.

SI and the Psychomotor Vigilance Task (PVT) are useful behavioral indicators of drinking-related risk. SI is a key indicator of acute alcohol response. Differences in sensitivity to alcohol's effects predict future heavy drinking and the risk of developing Alcohol use disorder (AUD) [4]. PVT is an indicator that is acutely sensitive to sleep deprivation and alcohol-induced transient impairment, as evidenced by reduced reaction time distributions and an increased lapse rate [5], and predicts the risk of motor vehicle accidents [6]. Abbreviated PVT protocols, including a 3-min version, validly detect alcohol-induced cognitive impairment and lapses and are considered highly informative in practical and operational settings [7]. These indicators therefore provide complementary measurements of the acute functional consequences of alcohol intake. At the same time, breath alcohol concentration (BrAC) functions as an objective measure of systemic alcohol exposure following drinking.

Alcohol is metabolized primarily via hepatic alcohol dehydrogenase (ADH) and aldehyde dehydrogenase (ALDH). Functional polymorphisms in *alcohol dehydrogenase 1B* (*ADH1B*; ADH1B*1 and ADH1B*2) and *aldehyde dehydrogenase 2* (*ALDH2*; ALDH2*1 and ALDH2*2) substantially influence the rate of alcohol elimination [8]. Individuals carrying ADH1B*2 or ALDH2*2 alleles exhibit accelerated ethanol oxidation or impaired acetaldehyde clearance, respectively, resulting in [8] greater accumulation and stronger aversive physiological responses during alcohol consumption [9]. Many studies in healthy adult males have shown no difference between the genotypes, ALDH2*1/*1 and *1/*2, in peak blood alcohol concentration (BAC) or BrAC trajectories, implying that the BAC/BrAC time course is largely independent of *ALDH2* genotype [9–11]. However, several studies—particularly in East Asian populations—have reported genotype-related differences in BrAC duration and elimination slope [12, 13]. Together, these findings suggest that the extent to which *ALDH2* genotype influences BrAC/BAC kinetics warrants re-examination.

While biological factors influence alcohol metabolism, repeated alcohol exposure can also lead to metabolic tolerance (enhanced metabolic capacity) and behavioral tolerance (reduced susceptibility to alcohol-induced impairment). The Alcohol Use Disorders Identification Test (AUDIT), which assesses the severity and risk of problem drinking, and the Alcohol Use Disorders Identification Test-Consumption (AUDIT-C), which evaluates alcohol consumption levels using three items from AUDIT, are widely used as indicators of drinking patterns [3, 14, 15]. A high AUDIT-C score is considered a threshold indicating potentially harmful drinking or alcohol use disorders [15]. Chronic heavy drinking may induce metabolic tolerance, partly via upregulation of hepatic alcohol-metabolizing enzymes, including cytochrome P450 family 2 subfamily E member 1 (CYP2E1) [16, 17]. Accordingly, BrAC/BAC profiles may vary across AUD/heavy drinkers (HD) and light drinkers (LD) groups due to combined effects of metabolic tolerance and absorption-related factors [16, 18]. In terms of behavioral tolerance, SI decreases with higher drinking tendency, though evidence is mixed regarding the association between habitual drinking and cognitive performance. A recent large-scale study suggests that HD and patients with AUD may exhibit less cognitive impairment and greater behavioral tolerance than LD [19]. This pattern raises the possibility of a mismatch between subjective intoxication and objective impairment.

In this context, biological factors such as genetic background and sex are known to influence drinking behavior. Individuals with an inactive ALDH2 enzyme encoded by the *2 allele tend to limit alcohol consumption because of unpleasant reactions (acetaldehyde accumulation), which consequently acts as a protective factor against alcohol dependence [8, 11]. In addition, female participants generally consume less alcohol and drink less frequently than male participants. Sex also influences BrAC levels; female participants typically have a lower body water content and reduced gastric first-pass metabolism, leading to higher BrAC levels after consuming the same amount of alcohol as male participants [16, 20]. The optimal AUDIT-C cutoff is also set lower for female participants (e.g., ≥ 3), and their score distributions tend to skew lower [21]. Together, these findings suggest that both genotype and sex shape drinking behavior and alcohol pharmacokinetics, and that drinking patterns may contribute to behavioral tolerance.

Clarifying how biological and behavioral factors jointly shape BrAC, subjective intoxication, and functional impairment is essential for understanding alcohol-related risk. To date, few studies have integrated genetic factors, drinking tendency, and their acute consequences (BrAC, SI, and PVT) within a single experimental framework. This study aimed to comprehensively evaluate variation in acute functional impairment and subjective perception during alcohol intoxication associated with *ADH1B*/*ALDH2* genotype and sex, considering two pathways: alcohol metabolism and drinking tendency. We quantitatively measured changes in SI and vigilance performance (PVT) over time following alcohol consumption and used structural equation modeling (SEM) to evaluate the contributions of BrAC, AUDIT-C, and genetic polymorphisms (*ADH1B*/*ALDH2*) to these measures.

Based on prior evidence, we tested the following hypotheses:

- Hypothesis 1: *ADH1B* polymorphisms are associated with BrAC, which in turn predicts SI and vigilance performance.
- Hypothesis 2: *ALDH2* polymorphisms are associated with drinking tendency (AUDIT-C), which in turn predicts SI and PVT lapse rates.

## Materials and methods

### Ethics statement

This study was approved by the Kyushu University Hospital Institutional Review Board (approval no. 24013). After the experimental procedures had been explained, written informed consent was obtained from all participants before enrollment. The study was registered in the UMIN Clinical Trials Registry (UMIN000055964).

### Participants

Healthy participants were recruited from Kyushu University and other nearby universities. A total of 104 participants (sex: male, n = 61; female, n = 43) participated in the screening process. Eligibility criteria were as follows: 1) Age ≥ 21 years, 2) BMI 18.0–30.0 kg/m^2^, 3) AUDIT score ≤ 14 points, 4) A history of consuming ≥ 50 g of pure alcohol in a day without clinically significant adverse health effects. Exclusion criteria included history of cardiovascular, renal, or hepatic disease; use of medications affecting sleep–wake cycles (e.g., first-generation antihistamines); shift work including night shifts; time zone travel (≥ 4 h) within the past month; sleep disorders; excessive caffeine intake (≥ 5 cups of coffee/day); and other factors determined by the physician. Participants showing a positive reaction on the alcohol patch test (Alcohol Constitution Test Patch, Life Care Giken Co., Ltd., Toyama, Japan) were also excluded. Accordingly, no participants with the ALDH2*2/*2 genotype were observed. This left a total of 98 eligible participants (sex: male, n = 58; female, n = 40).

### Study timeline

Pre-test instructions: Participants were instructed to avoid staying up late for three days prior to the experiment, refrain from drinking alcohol the day before, and get eight hours of sleep. Caffeine and smoking were prohibited on the day of the experiment. Participants were instructed to eat their usual lunch no later than five hours before the start of the experiment, and to drink only non-caffeinated beverages thereafter. Two hours before the start of the experiment, upon arrival at the laboratory, participants’ height and weight were measured, and saliva samples for genotyping were collected. Genotype determination was performed only after completion of all BrAC, SI, and PVT measurements. Thus, the experimenters were blind to the participants’ genotypes throughout the data collection process, minimizing the possibility of genotype-related experimenter bias during these measurements. Immediately afterwards, participants consumed a standardized light meal provided by the experimenters. Practice trials for the Go/No-go task and PVT were conducted before the main task to confirm participants understood the procedures. Following a 30-min break, baseline measurements were taken before drinking the alcohol, including SI assessment, BrAC measurement, and the main PVT trial.

**Fig. 1:**
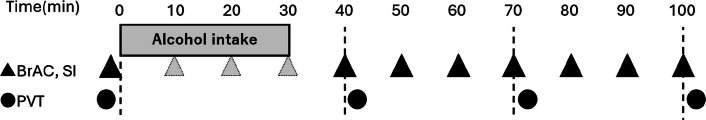
Experimental timeline. Participants began drinking at 0 min (time = 0). Breath alcohol concentration (BrAC) and subjective intoxication (SI) were assessed every 10 min from 0 to 100 min. The Psychomotor Vigilance Task (PVT) was administered at 0, 40, 70, and 100 min

After drinking began (0 min), a sequence of 11 BrAC/subjective measurements were taken, one every 10 min; participants were instructed to rinse their mouths and swallow a sip of water 3 min before each BrAC measurement. Data from 10 to 30 min were excluded from the analysis. In the lab, up to six participants performed the task side by side, separated by partitions. The PVT task was performed at 0, 40, 70, and 100 min (Fig. 1). After completing the measurements, participants were confirmed to have BrAC ≤ 0.10 mg/L. Following a nurse's health check, they were dismissed.

### DNA analysis

Saliva samples were collected using an Oragene Discover kit (OGR-600, DNA Genotek, Ottawa, Canada). Genomic DNA was then extracted from the saliva using the manufacturer's protocol with the PT-L2P-5 purification solution (DNA Genotek, Ottawa, Canada). The TaqMan SNP Genotyping Assay (Applied Biosystems, Waltham, MA, USA) was used to determine the genotypes of rs671 in *ALDH2* and rs1229984 in *ADH1B*. Polymerase chain reaction (PCR) was performed using the CFX96 real-time PCR system (Bio-Rad Laboratories, Hercules, CA, USA). The reactions were conducted using TaqMan Genotyping Master Mix (Thermo Fisher Scientific, Waltham, MA, USA) under the manufacturer-recommended conditions. The TaqMan probe AssayIDs were the following: C_11703892_10 (rs671 in *ALDH2*) and C_2688467_20 (rs1229984 in *ADH1B*). Genotype calling was performed automatically using CFX Manager, and the discrimination was also visually confirmed using the plot.

### Alcohol administration

Vodka at 0.5 g/kg body weight was administered orally in three separate doses 10 min apart, a total period of 30 min. The beverage was prepared by diluting 40% vodka to 12.5% with water and adding 8 g of lemon juice.

### Measurement indicators

BrAC was measured using the ST-3000 breath alcohol detector (Sanko Techno, Tokyo, Japan). At each time point, three consecutive measurements were taken and their mean value was used for analysis. The participants were instructed to rinse their mouths thoroughly before the measurements.

SI was assessed using a single-item visual analog scale (VAS) ranging from 0 (“not at all intoxicated”) to 100 (“very intoxicated”). At each measurement time point, participants rated their current level of subjective intoxication by adjusting a slider on a laptop screen using a mouse. The assessment form was implemented using Google Apps Script.

#### Psychomotor Vigilance Task (PVT)

To assess vigilance, subjects performed a 5-min PVT on a computer. Although the experimental protocol also included other cognitive tasks, such as a Go/No-go task, the present study focused on the PVT because the primary aim was to evaluate the temporal dynamics of vigilance impairment in relation to genetic factors and subjective perception. Stimuli were presented using Presentation (Neurobehavioral Systems, Berkeley, CA, USA). Inter-stimulus intervals were randomly set between two and ten seconds, and a numerical counter appeared at the center of the screen. Participants were instructed to press the spacebar as quickly as possible upon stimulus presentation. After each response, the reaction time (ms) was displayed as feedback. PVT performance was assessed using two metrics: lapse rate (%) and reciprocal reaction time (rRT; 1/RT). Lapse rate (%) was defined as the proportion of responses with reaction times exceeding 355 ms among valid trials; the count of such lapses (k) was divided by the number of valid trials (n) to obtain k/n. To avoid nonlinearity at the boundary values (0 or 1), lapse rate was analyzed on the logit scale after applying a small offset of (k + 0.5)/(n + 1). rRT was used as an index of reaction speed and was expressed in s⁻^1^ units. Participants with missing values at any time point were excluded from the analysis.

### Statistical analysis

A total of 98 participants were enrolled. Participants with the ADH1B*1/*1 genotype (n = 5) were not combined with ADH1B*1/*2 participants because the analytic plan treated *ADH1B* genotypes separately based on their functional differences. Because the ADH1B*1/*1 subgroup was very small, these participants were excluded from the primary analysis to avoid unstable estimates. Therefore, 93 participants (male, n = 55; female, n = 38) were included in the primary analysis.

#### Comparison of demographic characteristics

Table 1 shows the baseline characteristics of the participants. Sex (male/female) was compared among the four *ADH1B* genotype × *ALDH2* genotype groups using a chi-square test. Age, weight, height, BMI, and AUDIT-C were examined using a two-factor analysis of variance (ANOVA) (*ADH1B* × *ALDH2*).

**Table 1:** Participant characteristics by *ADH1B* × *ALDH2* genotype

		ALDH2*1/*1		ALDH2*1/*2	
	Overall	ADH1B*2/*2	*ADH1B* *1/*2	ADH1B*2/*2	*ADH1B* *1/*2
Characteristic	N = 93	N = 50	N = 15	N = 18	N = 10
Age	22.3 (1.6)	22.3 (1.1)	22.3 (1.0)	22.7 (3.3)	21.6 (0.7)
Sex, Female n (%)	38 (41%)	20 (40%)	9 (50%)	5 (33%)	4 (40%)
Height	166.9 (9.3)	167.3 (9.8)	164.1 (7.9)	167.2 (8.3)	169.3 (10.5)
Weight	58.1 (8.8)	58.9 (9.2)	55.4 (6.7)	57.1 (9.4)	60.3 (9.3)
BMI	20.8 (2.1)	21.0 (2.2)	20.5 (1.7)	20.3 (2.0)	21.0 (2.1)
AUDIT-C*	4.2 (1.9)	4.5 (1.8)	4.5 (2.1)	3.3 (2.0)	3.6 (1.6)

Values are mean (SD) unless otherwise stated. n indicates the number of participants

Sex was compared using Fisher’s exact test

^*^indicates a significant main effect of *ALDH2* (two-way ANOVA: *ADH1B* × *ALDH2*); for AUDIT-C, *p* = 0.0199. No significant main effects of *ADH1B* or interactions were observed

#### Comparison of BrAC, SI, and PVT performance

BrAC and SI were measured at ten-minute intervals between 0 and 100 min after drinking. For inferential statistics, a repeated- measures ANOVA was performed with time (8 levels: 0, 40–100 min) as the within-subjects factor and *ADH1B* and *ALDH2* genotype as between-subjects factors. PVT indices (lapse rate, rRT) were analyzed using time (4 levels: 0, 40, 70, 100 min) as a within-subject factor and *ADH1B* and *ALDH2* genotypes as between-subject factors. To address heterogeneity of variance, we used heteroskedasticity-consistent standard errors (HC3). When sphericity was violated, degrees of freedom based on the Greenhouse–Geisser correction were reported. Post hoc comparisons and graphical representations utilized estimated marginal means (EMMs) and 95% confidence intervals (CIs) for each condition, and Holm's method was applied for multiple comparison correction.

#### Structural equation modeling

SEM was conducted to examine the relationships among genotypes, drinking tendency, BrAC, SI, and PVT lapse rate. The model was specified as a hypothesis-driven observed-variable path model using measured variables only, rather than as a latent-variable model. In this framework, BrAC and AUDIT-C were included as intermediate variables representing acute alcohol exposure and habitual drinking tendency, respectively, rather than as formal biological mediators. Sex was coded as Female = 1, Male = 0; *ALDH2* genotype was coded as *1/*1 = 1, *1/*2 = 0; and *ADH1B* genotype was coded as *1/*2 = 1, *2/*2 = 0. All were entered as binary variables, with the reference category set to zero. Continuous variables (BrAC, SI, lapse, AUDIT-C) were z-standardized within each time point before analysis. As a hypothesis-based common model, we placed *ADH1B*, *ALDH*2, and sex upstream, AUDIT-C and BrAC as intermediate variables and SI and Δlapse rate (difference from the lapse rate at 0 min) downstream. The same model was applied separately at 40 min (near-peak) and 100 min (decline phase).

Specifically, the following structural equation model was assumed: first, AUDIT-C was predicted by *ALDH2*, sex, and *ADH1B*, and BrAC was predicted by *ALDH2*, AUDIT-C, sex, and *ADH1B*. As outcomes, SI was regressed on BrAC, AUDIT-C, and *ALDH2*, and lapse rate was regressed on BrAC, AUDIT-C, and *ALDH2*. The residual covariance between SI and lapse was freely estimated. Estimation used maximum likelihood with robust standard errors and a Satorra–Bentler scaled test statistic. All covariances between exogenous variables were freely estimated, and missing data were handled using full-information maximum likelihood. Model fit was assessed using the robust Satorra–Bentler chi-square statistic, the comparative fit index (CFI), Tucker–Lewis index (TLI), root mean square error of approximation (RMSEA), and standardized root mean square residual (SRMR), and standardized path coefficients with 95% CIs were reported.

#### Calculation of BrAC decline rate

A linear mixed-effects model was fitted with BrAC as the dependent variable, and time since drinking (minutes), centered at 80 min, as a continuous fixed effect. Random intercepts and slopes for each participant were included. Estimation used restricted maximum likelihood (REML), specifying bobyqa as the optimizer.

#### Statistical software

Analyses were performed using R software (version 4.4.3) [22] with the following packages: afex [23] for repeated-measures ANOVA with robust standard errors, emmeans [24] for estimated marginal means and post hoc comparisons, lavaan [25] for SEM, lme4 [26] for linear mixed-effects models, and dplyr [27], ggplot2 [28], and semPlot [29] for data processing and visualization. All statistical tests were two-tailed, with the significance level set at *p* < 0.05.

## Results

### Participant characteristics by genotype group

Analyses were conducted in 93 participants (male participants, n = 55; female participants, n = 38; mean age 22.3 ± 1.6 years) after excluding ADH1B*1/*1 genotype (n = 5), which was rare in the present sample. Participants were classified into four groups by the combination of *ADH1B* (*1/*2 vs *2/*2) and *ALDH2* (*1/*1 vs *1/*2) genotypes. AUDIT-C scores were significantly higher in ALDH2*1/*1 than in ALDH2*1/*2 (Table 1).

### Repeated-measures ANOVA

#### BrAC

**Fig. 2:**
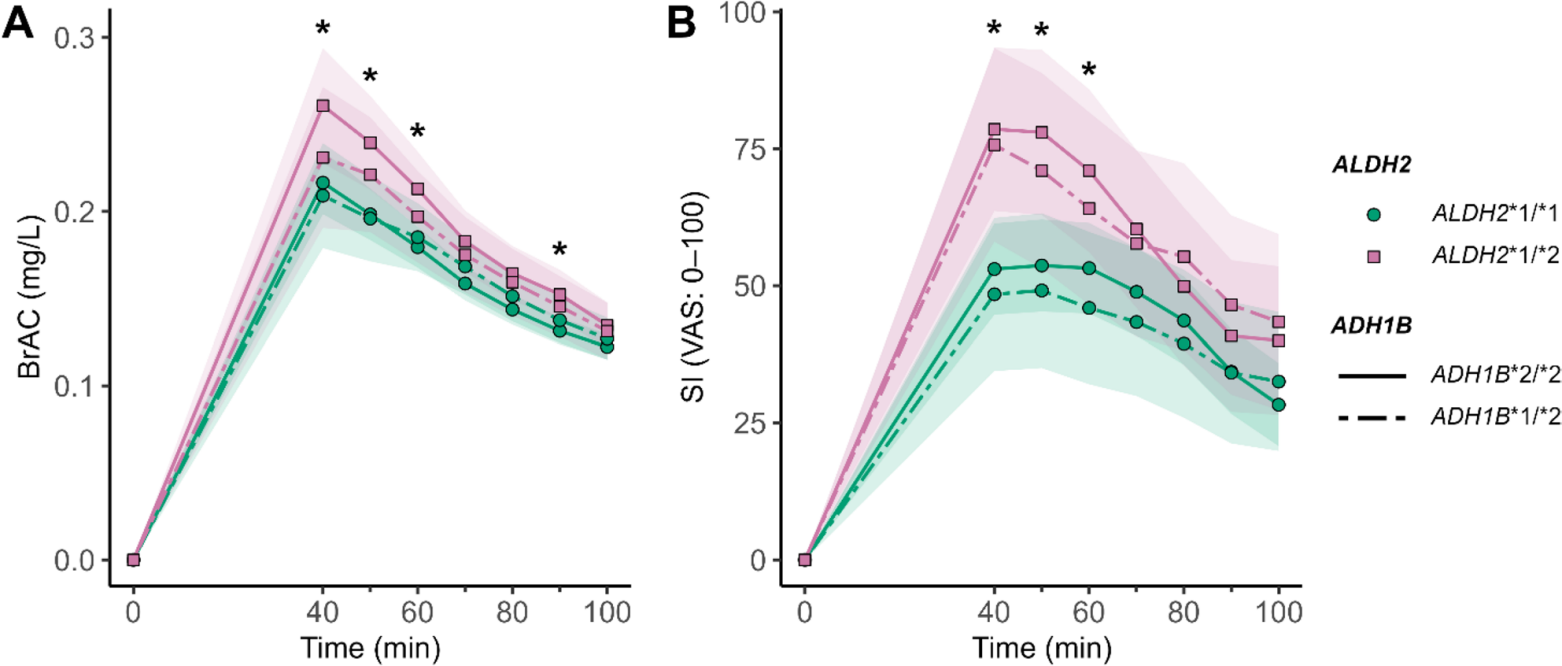
Time courses of breath alcohol concentration (BrAC) and subjective intoxication (SI) after alcohol consumption. **A** BrAC (mg/L) and **B** SI (VAS, 0–100) are shown across 0–100 min by *ADH1B* × *ALDH2* genotype group. Points indicate estimated marginal means (EMMs) and colored bands indicate 95% confidence intervals (CIs) from the repeated-measures ANOVA model (n = 93 for BrAC; n = 85 for SI). Asterisks indicate Holm-adjusted pairwise differences in *ALDH2* marginal means at each time point (*p* < 0.05)

BrAC was measured in all 93 participants. There was a significant *ALDH2* genotype × time interaction (F(1.73, 153.90) = 3.67, *p* = 0.034, partial *η*^2^ = 0.040; Fig. 2A). ALDH2*1/*1 showed lower BrAC than ALDH2*1/*2 at 40 (*p* = 0.040), 50 (*p* = 0.012), 60 (*p* = 0.030), and 90 min (*p* = 0.037), but not at 70, 80, or 100 min (p ≥ 0.059). There were also main effects of time (F(1.73, 153.90) = 562.09, *p* < 0.001, partial *η*^2^ = 0.863) and *ALDH2* (F(1, 89) = 5.17, *p* = 0.025, partial *η*^2^ = 0.055). No significant effects involving *ADH1B* were observed (Table 2). BrAC trajectories of the ADH1B*1/*1 participants are shown in Supplementary Fig. 1.

**Table 2:** Results of two-way ANOVA for the effects on BrAC and SI

	BrAC			SI		
Effect	*F *(df1, df2)	*p*-value	*η*_*p*_^2^	*F* (df1, df2)	*p*-value	*η*_*p*_^2^
*ADH1B*	0.26(1, 89)	0.613	0.003	0.1(1, 81)	0.759	0.001
*ALDH2*	5.17(1, 89)	0.025	0.055	6.54(1, 81)	0.012	0.075
*ADH1B*:*ALDH2*	0.80(1, 89)	0.373	0.009	0.04(1, 81)	0.846	< 0.001
time	562.09(1.73,153.9)	< 0.001	0.863	136.49(2.66, 215.08)	< 0.001	0.628
*ADH1B*:time	1.42(1.73,153.9)	0.244	0.016	1.27(2.66, 215.08)	0.287	0.015
*ALDH2*:time	3.67(1.73,153.9)	0.034	0.040	5.41(2.66, 215.08)	0.002	0.063
*ADH1B*:*ALDH2*:time	0.37(1.73,153.9)	0.657	0.004	0.30(2.66, 215.08)	0.801	0.004

*BrAC* breath alcohol concentration. SI: subjective intoxication

*F(df)* *F* statistic with (df1, df2); df are Greenhouse–Geisser corrected for within-subject effects

*ηp*^*2*^ partial eta squared

Tests: heteroskedasticity-consistent covariance estimator (HC3)

#### Subjective intoxication

SI was analyzed in 85 participants (eight excluded due to missing data). There was a significant *ALDH2* genotype × time interaction (F(2.66, 215.08) = 5.41, *p* = 0.002, partial *η*^2^ = 0.063; Fig. 2B). ALDH2*1/*1 reported lower SI than ALDH2*1/*2 at 40 (*p* < 0.001), 50 (*p* = 0.002), and 60 min (*p* = 0.013), but not from 70 to 100 min (*p* ≥ 0.063). There were also significant main effects of time (F(2.66, 215.08) = 136.49, *p* < 0.001, partial *η*^2^ = 0.628), and *ALDH2* (F(1, 81) = 6.54, *p* = 0.012, partial *η*^2^ = 0.075). No significant effects involving *ADH1B* were observed (Table 2).

#### PVT performance

**Fig. 3:**
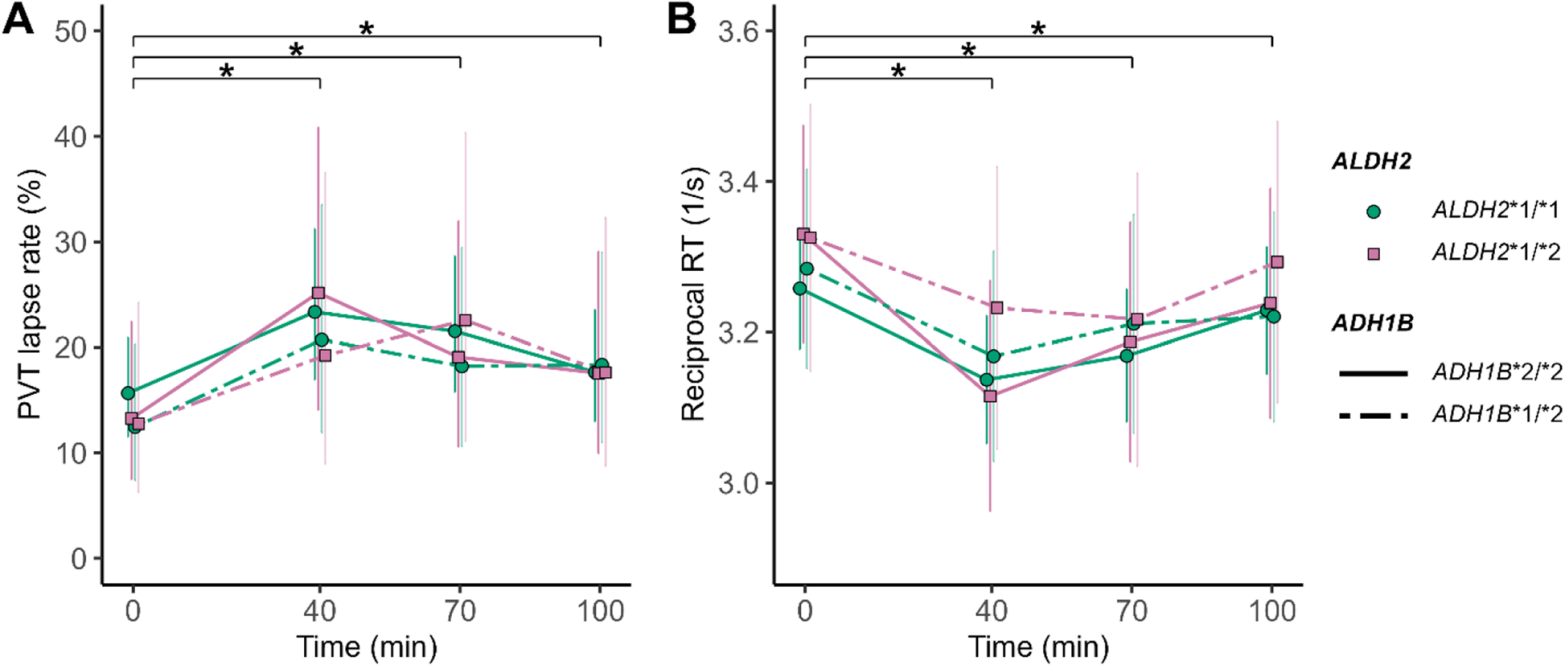
Time courses of PVT performance after alcohol consumption. **A** PVT lapse rate (%) and **B** reciprocal reaction time (rRT; 1/s) are shown across 0–100 min by *ADH1B* × *ALDH2* genotype group (n = 92). Points indicate estimated marginal means (EMMs) and error bars indicate 95% confidence intervals (CIs). In panel **A**, lapse rate (proportion of lapses > 355 ms on responded trials) was analyzed on the logit scale with a small offset and back-transformed to percentages. In panel **B**, rRT was calculated from responded trials excluding lapses > 500 ms. Brackets and asterisks indicate Holm-adjusted comparisons versus 0 min (*p* <.05)

PVT performance was analyzed in 92 participants (one excluded due to missing data). For lapse rate, there was a significant main effect of time (*F*(2.68, 235.78) = 9.26, *p* < 0.001, partial *η*^2^ = 0.095; Fig. 3A), with higher lapse rates at 40 (*p* < 0.001), 70 (*p* < 0.001), and 100 min (*p* = 0.010) than at 0 min. For rRT, there was also a significant main effect of time (*F*(2.58, 227.00) = 15.36, *p* < 0.001, partial *η*^2^ = 0.149; Fig. 3B), with lower rRT at 40 (*p* < 0.001), 70 (*p* < 0.001), and 100 min (*p* = 0.020) than at 0 min. No significant effects involving *ADH1B* or *ALDH2* genotype were observed for either measure (Table 3).

**Table 3:** Results of two-way ANOVA for the effects on PVT performance

	Lapse rate			rRT		
Effect	*F* (df1, df2)	*p*-value	*η*_*p*_^2^	*F* (df1, df2)	*p*-value	*η*_*p*_^2^
*ADH1B*	0.11 (1, 88)	0.745	0.001	0.28 (1, 88)	0.599	0.003
*ALDH2*	0.00 (1, 88)	0.966	< 0.001	0.23 (1, 88)	0.633	0.003
*ADH1B:ALDH2*	0.03 (1, 88)	0.861	< 0.001	0.04 (1, 88)	0.848	< 0.001
time	9.26 (2.68, 235.78)	< 0.001	0.095	15.36 (2.58, 227.00)	< 0.001	0.149
*ADH1B*: time	0.58 (2.68, 235.78)	0.607	0.007	0.82 (2.58, 227.00)	0.470	0.009
*ALDH2*: time	0.12 (2.68, 235.78)	0.933	0.001	0.44 (2.58, 227.00)	0.696	0.005
*ADH1B*: *ALDH2*: time	0.63 (2.68, 235.78)	0.577	0.007	0.88 (2.58, 227.00)	0.440	0.010

Lapse rate: proportion of lapses (>355 ms) analyzed on the logit scale with a small offset and back-transformed to percentages

rRT: reciprocal reaction time (1/s) excluding lapses >500 ms

*F(df)*: *F* statistic with (df1, df2); df are Greenhouse–Geisser corrected for within-subject effects

*η*_*p*_²: partial eta-squared

### SEM analysis

The path models were used to examine structured associations among genotype, sex, BrAC, AUDIT-C, SI, and PVT lapse rate at 40 and 100 min. Detailed estimates for all paths, including unstandardized coefficients, standard errors, 95% confidence intervals, standardized coefficients, and p-values, are provided in Supplementary Table 1.

#### 40-min model

**Fig. 4:**
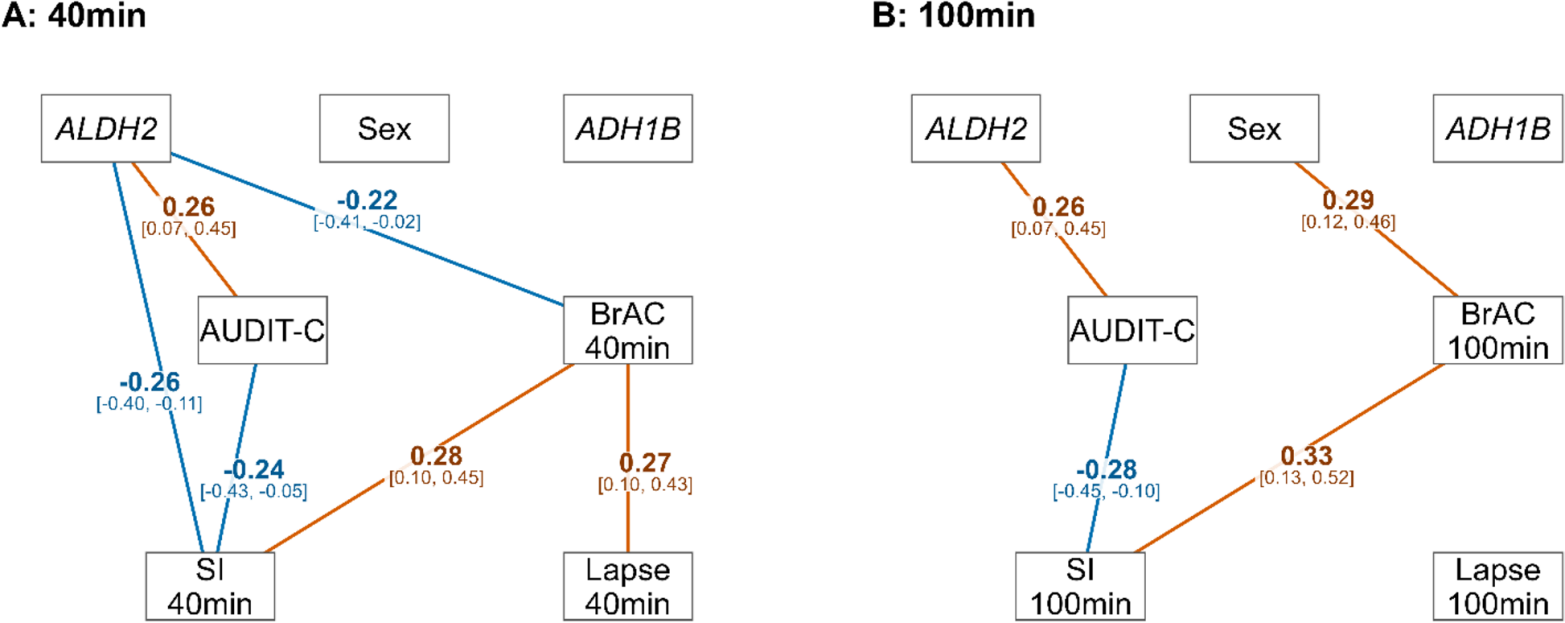
Common structural equation model fitted at 40 and 100 min after alcohol consumption. The same observed-variable path model structure was fitted at **A** 40 min and **B** 100 min to jointly model subjective intoxication (SI) and PVT lapse rate. Sex was coded as Female = 1 and Male = 0; *ALDH2* genotype was coded as 1 for *1/*1 and 0 for *1/*2; *ADH1B* genotype was coded as 1 for *1/*2 and 0 for *2/*2. Only statistically significant paths (two-sided α = 0.05) are shown. Arrow color indicates the sign of the path coefficient (positive = orange, negative = blue). Numbers on arrows indicate standardized path coefficients (*β*) with 95% confidence intervals in parentheses

The 40-min path model showed good fit (*χ*^2^(4) = 0.42, *p* = 0.98, CFI = 1.00, TLI = 1.31, RMSEA = 0.00 [90% CI 0.00–0.00], SRMR = 0.01; Fig. 4A). For downstream paths, higher BrAC was associated with higher SI (*β* = 0.28, *p* = 0.002) and higher lapse rate (*β* = 0.27, *p* = 0.001). Higher AUDIT-C was associated with lower SI (*β* = − 0.24, *p* = 0.013) but not with lapse rate (*β* = − 0.14, *p* = 0.11). For upstream paths, *ALDH2* genotype was positively associated with AUDIT-C (*β* = 0.26, *p* = 0.011) and negatively associated with BrAC (*β* = − 0.22, *p* = 0.033). *ALDH2* also showed a direct negative association with SI (*β* = − 0.26, *p* = 0.001), whereas the direct path to lapse rate was not significant (*β* = − 0.02, *p* = 0.87). No significant paths involving sex or *ADH1B* were observed.

#### 100-min model

The 100-min path model also showed good fit (*χ*^2^(4) = 2.93, *p* = 0.57, CFI = 1.00, TLI = 1.12, RMSEA = 0.00 [90% CI 0.00–0.14], SRMR = 0.02; Fig. 4B). For downstream paths, BrAC remained positively associated with SI (*β *= 0.33, *p* < 0.001), whereas its association with lapse rate was not significant (*β* = 0.08, *p* = 0.52). AUDIT-C was again negatively associated with SI (*β* = − 0.28, *p* = 0.002) but not with lapse rate (*β* = − 0.08, *p* = 0.53). For upstream paths, the positive association between *ALDH2* genotype and AUDIT-C persisted (*β* = 0.26, *p* = 0.011), whereas *ALDH2* was not significantly associated with BrAC (*β* = − 0.15, *p* = 0.15). Sex was positively associated with BrAC (*β* = 0.29, *p* = 0.002; Female = 1, Male = 0). Direct paths from *ALDH2* to SI (*β* = − 0.07, *p* = 0.48) and lapse rate (*β* = − 0.03, *p* = 0.80) were not significant. No significant paths involving *ADH1B* were observed. Several alternative SEMs were explored, but none improved model fit over the theoretically constrained main model.

### BrAC decline rate

BrAC decline rate was analyzed in all 93 participants. The mean decline rate was − 0.00152 mg/L/min (95% CI − 0.00173, − 0.00135), estimated from a linear mixed-effects model with BrAC as the dependent variable and time since drinking (minutes) as a continuous fixed effect, with random intercepts and slopes for each participant.

## Discussion

### Time course and genotype-related variation in BrAC and SI

In the present study, we examined associations of *ADH1B/ALDH2* genotypes and AUDIT-C with BrAC, SI, and PVT performance. Results from the repeated measures ANOVA showed that BrAC and SI declined over time after 40 min, whereas PVT performance deteriorated, consistent with prior vigilance task studies [2, 30].

Contrary to our expectation, *ADH1B* genotype was not associated with BrAC levels over time. The ADH1B*2 allele encodes an ADH enzyme with higher catalytic activity than ADH1B*1; however, because the difference in ethanol-to-acetaldehyde conversion activity between *1/*2 and *2/*2 is relatively small [11, 31, 32] and ADH1B*1/*1 was excluded, our analysis may have been underpowered to detect *ADH1B*-related BrAC differences. The BrAC trajectories of the excluded ADH1B*1/*1 participants are shown descriptively in Supplementary Fig. 1. Although the small sample size (n = 5) precludes formal inference, these participants tended to show higher BrAC than the ADH1B*1/*2 and *2/*2 groups, as expected from this functional difference, which leads to slower ethanol oxidation. This pattern indicated that ADH1B*1/*1 participants could not be appropriately combined with the *1/*2 group. We therefore excluded these participants from the formal analysis. By contrast, *ALDH2* genotype was significantly associated with BrAC. The ALDH2*2 allele reduces acetaldehyde oxidation, leading to acetaldehyde accumulation that may contribute to slower ethanol clearance and higher BrAC [13, 33]. Consistent with our results, a previous study reported a 1.96-fold higher ethanol clearance in Chinese individuals with ALDH2*1/*1 than in those with the ALDH2*1/*2 [12]. Thus, Hypothesis 1 was not supported: *ADH1B* genotype was not associated with BrAC in the present sample.

### Vigilance impairment and subjective–objective dissociation

Lapse rates and reaction times in the PVT test changed over time after alcohol administration, but no differences were observed between the genotypes. It should be noted that even at the 100-min mark, when the average BrAC fell below Japan's legal limit of 0.15 mg/L, performance was significantly impaired compared with baseline. This suggests that measurable vigilance impairment can persist into the decline phase after moderate alcohol intake. Genotype-related patterns may be more apparent for subjective experience than for simple performance measures at moderate doses.

Moreover, although acetaldehyde may impair cognitive function, many studies report little evidence for *ALDH2*-related differences under simple tasks or low alcohol doses (0.3–0.5 g/kg), and a Japanese study using facial flushing as a proxy for ALDH2*2/*2 genotype has reported greater subjective intoxication without differences in driving-related performance [34]. Together with our findings, this supports a clinically relevant dissociation: perceived intoxication and performance impairment do not necessarily align during alcohol intoxication.

### SEM integrates genotype, BrAC, and AUDIT-C in relation to SI

We used a hypothesis-driven observed-variable path model to integrate genotype, BrAC, and AUDIT-C in relation to SI and PVT lapse rates at 40 and 100 min. In this model, BrAC and AUDIT-C were treated as intermediate variables rather than formal biological mediators. This approach allowed us to examine conceptual links between genetic background, systemic alcohol exposure, habitual drinking tendency, and acute functional outcomes while maintaining a parsimonious model structure (Supplementary Table 1).

At 40 min, the model suggested three pathways linking the *ALDH2* genotype to SI. First, *ALDH2* genotype influenced SI indirectly through BrAC. Second, *ALDH2* genotype showed a direct path to SI. Specifically, participants with the ALDH2*1/*1 genotype reported lower SI. Compared with the ALDH2*1/*1 group, the ALDH2*1/*2 group has only 12–20% ALDH2 enzymatic activity, leading to elevated acetaldehyde levels and acute unpleasant reactions such as flushing and palpitations [9], which likely explains the higher SI reported by this group. Third, *ALDH2* genotype was associated with AUDIT-C, which in turn predicted SI. Individuals with ALDH2*1/*2 tend to restrict their chronic drinking because of the unpleasant reactions [31]. In this study, participants with ALDH2*1/*1 had significantly higher AUDIT-C scores than those with ALDH2*1/*2 (Table 1; Fig. 4A). However, AUDIT-C did not predict BrAC, contrary to our mediation hypothesis that the pathway from *ALDH2* genotype to BrAC is transmitted via AUDIT-C. This pattern is consistent with reports that AUDIT-C is not associated with physiological alcohol indices in healthy individuals [4]. In partial support of Hypothesis 2, *ALDH2* genotype was associated with AUDIT-C, and AUDIT-C predicted SI; however, AUDIT-C was not associated with BrAC or PVT outcomes.

Higher AUDIT-C predicted lower SI despite no association with BrAC or PVT lapse rate. Overall, our results suggest a discrepancy between self-reported intoxication and PVT performance. A prior study suggests that, within the normal-to-moderate range, differences in drinking tendency do not clearly affect vigilance function [35]. Consistent with our eligibility criterion (AUDIT ≤ 14), our results likewise indicate that, within this range, drinking tendency influence SI more than vigilance.

The discrepancy between subjective perception and performance provides insight into the mechanisms underlying risk perception and overconfidence during drinking. Contrary to our initial hypothesis, drinking tendency affected neither BrAC nor vigilance but only SI, implying that routine drinking within the normal range may be insufficient to modify metabolic rate or behavioral tolerance and instead primarily alters self-assessment.

In the 40-min model, a 1-point higher AUDIT-C (i.e., a one-category shift in drinking frequency or quantity) was associated with a 4.5-point lower SI, comparable to the SI difference associated with approximately 0.031 mg/L lower BrAC. Based on the average BrAC decline rate during the decline phase (40–100 min; − 0.00152 mg/L/min), this corresponds to roughly 20 min of the average BrAC decline (95% CI 8.7–48.3). This comparison is descriptive rather than causal. Nevertheless, it suggests that even modest differences in drinking tendency may translate into meaningful differences in perceived sobriety, potentially affecting decisions such as continued drinking or returning home. Field studies in bar settings have consistently reported that individuals with higher AUDIT-C scores show higher BrAC and are more likely to consume driving-impairing amounts, even when they are designated drivers [36, 37].

The dose in this study was 0.5 g/kg, a moderate amount. To ensure safety, it was administered over a period of 30 min, with water intake every 10 min. Compared with the high doses of 0.6 g/kg or more frequently used in previous studies, the peak BrAC was lower. Some studies have suggested that significant differences in psychomotor function between the two genotype groups were detected only after high-dose alcohol administration exceeding 0.5 g/kg [10, 13, 35]. Although vigilance impairment was detected in the Matsuura et al*.* study, the moderating roles of genotype and drinking tendency may not have been sufficiently evident. Even considering these findings, our results indicate that BrAC, which varied by *ALDH2* genotype, serves as a common basis for both SI and vigilance performance, whereas drinking tendency was related only to SI. This pattern may help explain discrepancies between psychological states and behavioral performance under the influence of alcohol.

In this context, the SEM results indicated that the salience of predictors differed across outcomes and time points. The association of AUDIT-C with SI persisted at 100 min, and *ALDH2* genotype remained indirectly linked to SI, whereas none of the hypothesized factors were significantly associated with vigilance at that time point. For BrAC, association with *ALDH2* genotype association was stronger at 40 min but weakened by 100 min.

### Limitations

This study has several limitations. Participants were limited to young, healthy adults of East Asian ancestry, so caution is needed when generalizing the results to populations that differ in age, ethnicity, or drinking patterns. Because the observation period was limited to 100 min, the present study could not evaluate the complete recovery phase as BrAC continued to decline toward zero. In addition, measurements obtained at later time points may be influenced not only by declining alcohol effects but also by fatigue or the repeated-testing effects inherent in the protocol. A longer observation period with an optimized measurement schedule may help clarify whether the dissociation between SI and vigilance performance persists or changes during the further decline in BrAC.

SEM was conducted at only two representative time points and therefore did not capture the full dynamics of change. Because of power constraints, we did not model complex interactions or include additional covariates. Therefore, these path models should be interpreted as hypothesis-driven summaries of structured associations rather than as confirmatory evidence for causal mediation. In addition, we did not adjust for variables that may affect PVT performance, such as sleepiness or fatigue. Finally, the functional assessment in this study was primarily centered on vigilance using the PVT, because the primary aim was to repeatedly assess this specific domain during acute alcohol exposure using a brief and well-established task. Therefore, other cognitive functions relevant to real-life situations, such as inhibitory control, divided attention, or more complex task performance, were not evaluated in the present analysis.

Although sex was included as a covariate in the path model, the present study was not specifically designed to examine sex differences. While our model identified a significant association between sex and BrAC during the decline phase, no significant direct paths from sex to SI or PVT lapse rate were observed.

In addition, factors that may contribute to sex-related variation in alcohol responses, such as body composition, body water distribution, and menstrual cycle status, were not controlled. Therefore, these sex-related findings should be interpreted with caution and should not be taken as evidence for specific physiological mechanisms.

Future studies should target broader age ranges, more diverse genotypes (particularly including ADH1B*1/*1), or clinical samples, and use multiple alcohol doses and more complex cognitive tasks to characterize gene–environment interactions more precisely.

## Conclusions

This study comprehensively examined variation in BrAC, SI, and vigilance task performance during alcohol intoxication associated with *ADH1B*/*ALDH2* genotypes and AUDIT-C score within a single modeling framework. The results indicate that BrAC was related to both SI and impaired vigilance function; conversely, drinking tendency was not associated with BrAC itself but only was related to self-reported SI and was not significantly associated with vigilance function. This suggests that while drinking tendency may reinforce the sensation of "not being drunk," the actual decline in cognitive performance may progress to the same degree regardless of drinking tendency. Furthermore, the *ALDH2* genotype had a significant association with BrAC, revealing that differences in metabolic capacity may be indirectly linked to subjective and behavioral outcomes through BrAC. These findings underscore the importance of considering subjective and objective measures when interpreting the psychological and behavioral effects of alcohol. They also highlight how genetic factors and drinking tendency jointly shape these outcomes.

## Supplementary Information

Supplementary Material 1.

## Data Availability

All datasets generated and/or analyzed in this study are available from the corresponding authors upon reasonable request.

## Abbreviations

*ADH1B*: *Alcohol dehydrogenase 1B*
*ALDH2*: *Aldehyde dehydrogenase 2*
ANOVA: Analysis of variance
AUD: Alcohol use disorder
AUDIT: Alcohol Use Disorders Identification Test
AUDIT-C: Alcohol Use Disorders Identification Test—Consumption
BAC: Blood alcohol concentration
BrAC: Breath alcohol concentration
CFI: Comparative fit index
CI: Confidence interval
HD: Heavy drinkers
LD: Light drinkers
*η*_*p*_^2^: Partial eta squared
PVT: Psychomotor Vigilance Task
RMSEA: Root mean square error of approximation
rRT: Reciprocal reaction time
SE: Standard error
SEM: Structural equation modeling
SI: Subjective intoxication
SRMR: Standardized root mean square residual
TLI: Tucker–Lewis index
VAS: Visual analog scale
*β*: Standardized path coefficient

## References

[1] World Health Organization. Global status report on alcohol and health 2018. World Health Organization; 2018. Accessed 18 Oct 2025.

[2] Moskowitz H, Florentino D. A review of the literature on the effects of low doses of alcohol on driving-related skills (Report No. DOT-HS-809-028). National Highway Traffic Safety Administration. 10.21949/15254682000.

[3] Fillmore MT, Ostling EW, Martin CA, Kelly TH. Acute effects of alcohol on inhibitory control and information processing in high and low sensation-seekers. Drug Alcohol Depend. 2009;100(1–2):91–9. 19004578 10.1016/j.drugalcdep.2008.09.007 PMC2647687

[4] Bramness JG, Skulberg KR, Skulberg A, Moe JS, Mørland J. The self-rated effects of alcohol are related to presystemic metabolism of alcohol. Alcohol Alcohol. 2023;58(2):203–8. 36715301 10.1093/alcalc/agad002 PMC10008105

[5] Howard ME, Jackson ML, Kennedy GA, Swann P, Barnes M, Pierce RJ. The interactive effects of extended wakefulness and low-dose alcohol on simulated driving and vigilance. Sleep. 2007;30(10):1334–40. 17969467 10.1093/sleep/30.10.1334 PMC2266271

[6] Matsuo R, Tanigawa T, Oshima A, Tomooka K, Ikeda A, Wada H, et al. Decreased psychomotor vigilance is a risk factor for motor vehicle crashes irrespective of subjective daytime sleepiness: the Toon Health Study. J Clin Sleep Med. 2023;19(2):319–25. 36271594 10.5664/jcsm.10328 PMC9892751

[7] Benderoth S, Hörmann HJ, Schießl C, Elmenhorst EM. Reliability and validity of a 3-min psychomotor vigilance task in assessing sensitivity to sleep loss and alcohol: fitness for duty in aviation and transportation. Sleep. 2021;44(11):zsab151. 34137863 10.1093/sleep/zsab151

[8] Edenberg HJ. The genetics of alcohol metabolism: role of alcohol dehydrogenase and aldehyde dehydrogenase variants. Alcohol Res Health. 2007;30(1):5–13. 17718394 PMC3860432

[9] Wall TL, Luczak SE, Hiller-Sturmhöfel S. Biology, genetics, and environment: underlying factors influencing alcohol metabolism. Alcohol Res Curr Rev. 2016;38(1):59–68. 10.35946/arcr.v38.1.08 PMC4872614 27163368

[10] Shin HY, Shin IS, Yoon JS. ALDH2 genotype-associated differences in the acute effects of alcohol on P300, psychomotor performance, and subjective response in healthy young Korean men: a double-blind placebo-controlled crossover study. Hum Psychopharmacol Clin Exp. 2006;21(3):159–66. 10.1002/hup.755 16565959

[11] Chen YC, Yang LF, Lai CL, Yin SJ. Acetaldehyde enhances alcohol sensitivity and protects against alcoholism: evidence from alcohol metabolism in subjects with variant ALDH2*2 gene allele. Biomolecules. 2021;11(8):1183. 34439848 10.3390/biom11081183 PMC8391449

[12] Ye Y, Lin Y, Haseba T, Chen F, Cui F, Yi X, et al. Sex- and ALDH2-dependent differences in alcohol metabolism and psychomotor performance: a study in Han Chinese adults after binge drinking. Ann Med. 2025;57(1):2496798. 40289679 10.1080/07853890.2025.2496798 PMC12039403

[13] Matsuura K, Kushio S, Imai H, Kudo H, Wakuda H, Otani N, et al. Association between psychomotor function and ALDH2 genotype after consuming barley shochu: a randomized crossover trial. Clin Transl Sci. 2023;16(4):686–93. 36748664 10.1111/cts.13482 PMC10087066

[14] Reinert DF, Allen JP. The Alcohol Use Disorders Identification Test: an update of research findings. Alcohol Clin Exp Res. 2007;31(2):185–99. 17250609 10.1111/j.1530-0277.2006.00295.x

[15] Bradley KA, DeBenedetti AF, Volk RJ, Williams EC, Frank D, Kivlahan DR. AUDIT-C as a brief screen for alcohol misuse in primary care. Alcohol Clin Exp Res. 2007;31(7):1208–17. 17451397 10.1111/j.1530-0277.2007.00403.x

[16] Cederbaum AI. Alcohol metabolism. Clin Liver Dis. 2012;16(4):667–85. 23101976 10.1016/j.cld.2012.08.002 PMC3484320

[17] Ueno F, Matsushita S, Hara S, Oshima S, Roh S, Ramchandani VA, et al. Influence of alcohol and acetaldehyde on cognitive function: findings from an alcohol clamp study in healthy young adults. Addiction. 2022;117(4):934–45. 34735038 10.1111/add.15733

[18] Winek CL, Murphy KL. The rate and kinetic order of ethanol elimination. Forensic Sci Int. 1984;25(3):159–66. 6745819 10.1016/0379-0738(84)90189-0

[19] Didier N, Vena A, Feather AR, Grant JE, King AC. Holding your liquor: comparison of alcohol-induced psychomotor impairment in drinkers with and without alcohol use disorder. Alcohol Clin Exp Res. 2023;47(6):1156–66. 10.1111/acer.15080 37330919

[20] Frezza M, Di Padova C, Pozzato G, Terpin M, Baraona E, Lieber CS. High blood alcohol levels in women. N Engl J Med. 1990;322(2):95–9. 2248624 10.1056/NEJM199001113220205

[21] Duffy FF, Sudom K, Jones M, Fear NT, Greenberg N, Adler AB, et al. Calibrating the Alcohol Use Disorders Identification Test-Consumption (AUDIT-C) for detecting alcohol-related problems among Canadian, UK and US soldiers: cross-sectional pre-deployment and post-deployment survey results. BMJ Open. 2023;13(5):e068619. 37130676 10.1136/bmjopen-2022-068619 PMC10163557

[22] R Core Team. R: A language and environment for statistical computing. Vienna: R Foundation for Statistical Computing; 2025. Available from: https://www.r-project.org/ Accessed 10 Dec 2025.

[23] Singmann H, Bolker B, Westfall J, Aust F, Ben-Shachar MS. afex: Analysis of factorial experiments. R package version 1.5–1; 2025. Available from: https://CRAN.R-project.org/package=afex. Accessed 10 Dec 2025.

[24] Lenth RV, Piaskowski J. emmeans: Estimated marginal means, aka least-squares means. R package version 2.0.1; 2025. Available from: https://CRAN.R-project.org/package=emmeans. Accessed 10 Dec 2025.

[25] Rosseel Y. lavaan: An R package for structural equation modeling. J Stat Softw. 2012;48(2):1–36.

[26] Bates D, Mächler M, Bolker B, Walker S. Fitting linear mixed-effects models using lme4. J Stat Softw. 2015;67(1):1–48.

[27] Wickham H, François R, Henry L, Müller K, Vaughan D, et al. dplyr: A grammar of data manipulation. R package version 1.1.4; 2023. Available from: https://CRAN.R-project.org/package=dplyr. Accessed 10 Dec 2025.

[28] Wickham H. Create elegant data visualisations using the grammar of graphics. 2016. Available from: https://ggplot2.tidyverse.org/. Accessed 10 Dec 2025.

[29] Epskamp S, Stuber S, Nak J, Veenman M, Jorgensen TD. semPlot: Path diagrams and visual analysis of various SEM packages’ output. R package version 1.1.7; 2025. Available from: https://CRAN.R-project.org/package=semPlot. Accessed 10 Dec 2025.

[30] Elmenhorst EM, Elmenhorst D, Benderoth S, Kroll T, Bauer A, Aeschbach D. Cognitive impairments by alcohol and sleep deprivation indicate trait characteristics and a potential role for adenosine A1 receptors. Proc Natl Acad Sci U S A. 2018;115(31):8009–14. 30012607 10.1073/pnas.1803770115 PMC6077699

[31] Oshima S, Haseba T, Masuda C, Kakimi E, Sami M, Kanda T, et al. Individual differences in blood alcohol concentrations after moderate drinking are mainly regulated by gastric emptying rate together with ethanol distribution volume. Food Nutr Sci. 2012;3(6):732–7.

[32] Tóth R, Fiatal S, Petrovski B, McKee M, Ádány R. Combined effect of ADH1B rs1229984, rs2066702 and ADH1C rs1693482/rs698 alleles on alcoholism and chronic liver diseases. Dis Markers. 2011;31(5):267–77. 22048268 10.3233/DMA-2011-0828 PMC3826929

[33] Crabb DW, Matsumoto M, Chang D, You M. Overview of the role of alcohol dehydrogenase and aldehyde dehydrogenase and their variants in the genesis of alcohol-related pathology. Proc Nutr Soc. 2004;63(1):49–63. 15099407 10.1079/pns2003327

[34] Fujita G, Nishida Y. Effect of a small dose of alcohol on driving-related behavior of Japanese who self-reported facial flush and nonflushing. Percept Mot Skills. 2009;109(3):651–63. 20178264 10.2466/pms.109.3.651-663

[35] Brumback T, Cao D, King A. Effects of alcohol on psychomotor performance and perceived impairment in heavy binge social drinkers. Drug Alcohol Depend. 2007;91(1):10–7. 17560739 10.1016/j.drugalcdep.2007.04.013 PMC2764986

[36] Barry AE, Chaney BH, Stellefson ML. Breath alcohol concentrations of designated drivers. J Stud Alcohol Drugs. 2013;74(4):509–13. 23739013 10.15288/jsad.2013.74.509 PMC3711342

[37] Martin RJ, Chaney BH, Cremeens-Matthews J. Examination of breath alcohol concentration (BrAC) levels, alcohol use disorders identification test (AUDIT-C) classification, and intended plans for getting home among bar-attending college students. Am J Addict. 2015;24(4):285–8. 25823777 10.1111/ajad.12196

