# Supplementary material for: Discrepancy between self-reported intoxication and psychomotor vigilance task performance after alcohol intake: associations with BrAC, alcohol-metabolizing genotypes, and drinking tendency
Source: J Physiol Anthropol. 2026 Jun 10;45:19. doi: 10.1186/s40101-026-00434-w (PMC13474664; doi:10.1186/s40101-026-00434-w)
Supplement: Supplementary file 1 [file 40101_2026_434_MOESM1_ESM.docx]

 **Supplementary Materials**

Supplementary Figure 1. BrAC time courses by *ADH1B* and *ALDH2* genotype, including the *ADH1B* *1/*1 participants. The BrAC trajectories of the five participants with *ADH1B* *1/*1 are shown separately for descriptive purposes, as this subgroup was too small for formal statistical analysis. Thick lines indicate estimated marginal means (EMMs), and thin lines indicate individual trajectories. Circles indicate *ALDH2* *1/*1 and squares indicate *ALDH2* *1/*2.

| Supplementary Table 1. Path coefficient estimates for the path models at 40 and 100 min after alcohol intake. | | | | | | |  |
| --- | --- | --- | --- | --- | --- | --- | --- |
| Time | Path | *B* | SE | 95% CI for *B* | *β* | 95% CI for　β | *p* |
| 40 min | ***ALDH2*(*1/*1 vs *1/*2) -> AUDIT-C** | **0.576** | **0.226** | **0.132, 1.019** | **0.257** | **0.068, 0.445** | **0.011** |
|  | ***ALDH2*(*1/*1 vs *1/*2) -> BrAC** | **-0.489** | **0.229** | **-0.938, -0.040** | **-0.218** | **-0.412, -0.024** | **0.033** |
|  | ***ALDH2*(*1/*1 vs *1/*2) -> SI** | **-0.574** | **0.170** | **-0.908, -0.240** | **-0.256** | **-0.398, -0.114** | **<0.001** |
|  | *ALDH2*(*1/*1 vs *1/*2) -> PVT lapse | -0.037 | 0.219 | -0.465, 0.391 | -0.016 | -0.206, 0.173 | 0.866 |
|  | Sex (Female vs male) -> AUDIT-C | -0.309 | 0.195 | -0.691, 0.072 | -0.153 | -0.336, 0.031 | 0.112 |
|  | Sex (Female vs male) -> BrAC | 0.245 | 0.206 | -0.160, 0.649 | 0.121 | -0.078, 0.320 | 0.235 |
|  | *ADH1B* (*1/*2 vs *2/*2) -> AUDIT-C | 0.078 | 0.227 | -0.367, 0.524 | 0.036 | -0.168, 0.240 | 0.731 |
|  | *ADH1B* (*1/*2 vs *2/*2) -> BrAC | -0.234 | 0.189 | -0.605, 0.137 | -0.108 | -0.275, 0.059 | 0.216 |
|  |  |  |  |  |  |  |  |
|  | AUDIT-C -> BrAC | -0.150 | 0.094 | -0.334, 0.033 | -0.150 | -0.338, 0.037 | 0.108 |
|  | **AUDIT-C -> SI** | **-0.243** | **0.098** | **-0.436, -0.051** | **-0.243** | **-0.432, -0.055** | **0.013** |
|  | AUDIT-C -> PVT lapse | -0.138 | 0.087 | -0.308, 0.032 | -0.138 | -0.300, 0.025 | 0.113 |
|  | **BrAC -> SI** | **0.275** | **0.091** | **0.097, 0.453** | **0.275** | **0.104, 0.446** | **0.002** |
|  | **BrAC -> PVT lapse** | **0.269** | **0.081** | **0.110, 0.428** | **0.269** | **0.105, 0.433** | **<0.001** |
|  |  |  |  |  |  |  |  |
| 100 min | ***ALDH2*(*1/*1 vs *1/*2) -> AUDIT-C** | **0.576** | **0.226** | **0.132, 1.019** | **0.257** | **0.068, 0.445** | **0.011** |
|  | *ALDH2*(*1/*1 vs *1/*2) -> BrAC | -0.338 | 0.236 | -0.801, 0.124 | -0.151 | -0.350, 0.049 | 0.152 |
|  | *ALDH2*(*1/*1 vs *1/*2) -> SI | -0.148 | 0.210 | -0.558, 0.263 | -0.066 | -0.250, 0.118 | 0.481 |
|  | *ALDH2*(*1/*1 vs *1/*2) -> PVT lapse | -0.063 | 0.249 | -0.551, 0.424 | -0.028 | -0.244, 0.188 | 0.799 |
|  | Sex (Female vs male) -> AUDIT-C | -0.309 | 0.195 | -0.691, 0.072 | -0.153 | -0.336, 0.031 | 0.112 |
|  | **Sex (Female vs male) -> BrAC** | **0.593** | **0.187** | **0.226, 0.959** | **0.293** | **0.124, 0.462** | **0.002** |
|  | *ADH1B* (*1/*2 vs *2/*2) -> AUDIT-C | 0.078 | 0.227 | -0.367, 0.524 | 0.036 | -0.168, 0.240 | 0.731 |
|  | *ADH1B* (*1/*2 vs *2/*2) -> BrAC | 0.039 | 0.186 | -0.325, 0.404 | 0.018 | -0.150, 0.186 | 0.833 |
|  |  |  |  |  |  |  |  |
|  | AUDIT-C -> BrAC | -0.131 | 0.098 | -0.324, 0.062 | -0.131 | -0.333, 0.071 | 0.182 |
|  | **AUDIT-C -> SI** | **-0.276** | **0.091** | **-0.454, -0.098** | **-0.277** | **-0.450, -0.104** | **0.002** |
|  | AUDIT-C -> PVT lapse | -0.082 | 0.129 | -0.335, 0.171 | -0.082 | -0.328, 0.165 | 0.527 |
|  | **BrAC -> SI** | **0.327** | **0.091** | **0.149, 0.504** | **0.327** | **0.134, 0.521** | **<0.001** |
|  | BrAC -> PVT lapse | 0.074 | 0.116 | -0.153, 0.301 | 0.074 | -0.160, 0.308 | 0.522 |
| Note. B: unstandardized path coefficient; SE: standard error; CI: confidence interval; β: standardized path coefficient; p: p-value. Statistically significant coefficients (p < .05) are indicated in bold. Sex was coded as 1 for female and 0 for male; *ALDH2* genotype was coded as 1 for *1/*1 and 0 for *1/*2; *ADH1B* genotype was coded as 1 for *1/*2 and 0 for *2/*2. All continuous variables (BrAC, SI, lapse, and AUDIT-C) were z-standardized before analysis. | | | | | | | |
